# Towards an equitable internship programme at WHO: is reform nigh?

**DOI:** 10.1136/bmjgh-2016-000088

**Published:** 2016-09-23

**Authors:** Ashton Barnett-Vanes, Cheng Feng, Maziar Jamnejad, Jing Jun

**Affiliations:** 1 Faculty of Medicine, Imperial College London, London, UK; 2 Public Health Research Centre, Tsinghua University, Beijing, China; 3 London, UK; 4 School of Social Sciences, Tsinghua University, Beijing, China

**Keywords:** Health policies and all other topics

**Summary Box**

- The WHO internship programme is one of the most high-profile junior professional training programme in global health, and previous findings have suggested the programme is inaccessible to young professionals from developing countries. However, the extent of this is unknown.
- In May 2016, WHO published, for the first time, full statistics concerning Member State participation on the internship programme – they show that only 15% of interns at WHO headquarters were from developing countries; Africa and South-East Asia regional offices offer less than 4% of all WHO internships; and almost 60% of WHO Member States had no nationals participating in the entire programme throughout 2015.
- The internship programme suffers from inequitable Member State participation, and therefore fails to build future global health capacity in young professionals from developing countries.
- Reform of the internship programme should focus on overhauling the entire recruitment procedures; providing financial support to interns, particularly from low-income countries; and introducing a semi-structured curriculum to maximise the benefits of the internship.

The global health workforce is under immense strain.^1^ For example, Africa—a continent with one-third of the world's disease burden has only ∼3% of global health personnel.^2^ This year WHO launched its *Global Strategy on Human Resources for Health*^3^ (GSHRH)—calling for a redoubling of efforts to better train and equip the global health workforce in order to strengthen public health capacity; echoing the 2006 World Health Assembly (WHA) resolution ‘Rapid scaling up of health workforce production’ passed in 2006.^4^

In pursuit of these aims, WHO runs a range of external programmes to train public health professionals, including the WHO Fellowship Programme; and through partnership with over 700 collaborating centres—often at universities—in more than 80 countries. In addition to these external programmes, WHO also runs *what should be* an internal training programme for future public health leaders: the WHO internship programme. Now in its 50th year, the programme has recently come under increased scrutiny—catalysed by research published in 2014 that suggested it was inaccessible to young professionals from developing countries.^5^ With both civil society and Member States recognising the potentially corrosive impact of a high-profile internal training programme that contradicts core global health policy;^6^ the debate surrounding the programme has intersected the wider issue of developing country engagement in international health training opportunities and governance. This commentary presents an overview of the challenges and progress towards making WHO's internship programme equitable.

## The internship programme

WHO is pre-eminent in global health; central to its mandate is a responsibility to support all 194 of its Member States in addressing their health challenges. By offering ‘concrete professional experience in an international environment’^7^ to young public health professionals through internships, WHO realises its commitment to states’ future, as well as current, health capabilities. These internships last 3–6 months; they are offered at WHO's headquarters, regional and certain country offices and provide unique exposure and training in global public health. Moreover, through networking opportunities and such else, these internships serve as a key point of career entry for many junior public health professionals.

## First signs of trouble

However, the findings published in 2014 cast a shadow over these ambitions—only 5% of headquarters interns were living in a developing country;^5^ corroborating the 2009 United Nations Joint Inspection Unit report which found 60% of interns on the WHO internship programme were from just five developed countries.^9^ What good is offering early career stage public health training, if young professionals from developing countries—where disease burdens and skills shortages are highest—cannot access them? Instead, these opportunities are taken up by nationals from a small number of high-income countries, which have health or academic institutions with long-standing relationships with WHO staff or departments, for reasons explained shortly. For example, in summer 2013 50% of interns at the WHO headquarters’ programme hailed from just two high-income countries.^5^ Despite the evidence, WHO was slow to act. It did not publish statistics to corroborate or refute the findings, and no statement or comment was offered on how it hoped to address the issues raised. Indeed, in 2013 and 2014 the internship programme was not mentioned in its human resources annual report.

## WHO's governing bodies

In January 2015, a written statement was submitted by a group of civil society and non-governmental organisation members to the human resources session of the WHO Executive Board.^10^ This raised concerns about the imbalance in Member State representation on the internship programme, drawing attention to the now mounting evidence. However, it was not until the involvement of WHO's governing bodies in January 2016 that WHO published—for the first time—some of its own centrally collected internship programme statistics. They were stark: among its ∼1000 annual internships offered worldwide in 2014, only 20% were undertaken by candidates from developing countries; the African regional office offered <2% of all WHO internships.^11^ These results confirmed what many feared—Member State representation on the internship programme was not equitable, nor was it driven by a clear policy objective. At the January 2016 Executive Board, a number of Member States expressed views on the implications of these findings; however, key information concerning the national origin of interns was not released at the time,^6^ frustrating efforts to achieve consensus on the action to be taken.

**Figure 1:**
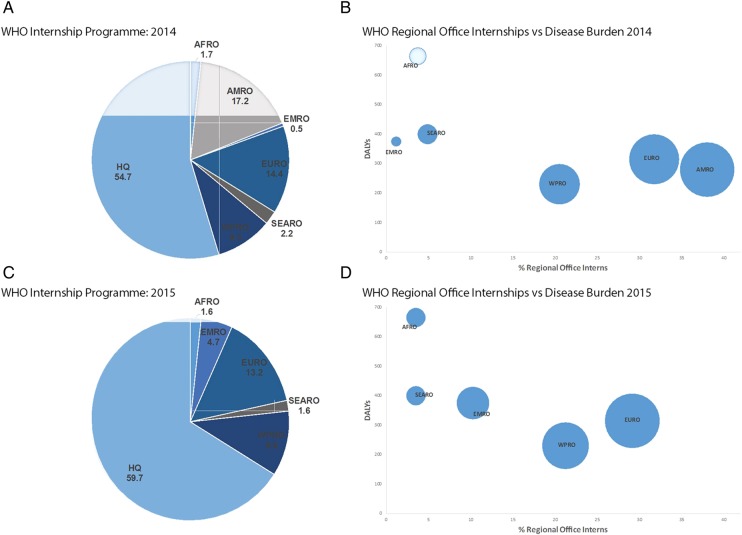
WHO-IP and regional office opportunities in 2014 and 2015. (A) Distribution (%) of WHO internships across headquarters and regional offices in 2014 and 2015. (B) WHO-IP regional office internship opportunities against regional disease burden disability-adjusted life years (DALYs); circle size denotes proportion (%) of all regional opportunities (same as X-axis). HQ, headquarters; AFRO, African regional office; AMRO*, regional office of the Americas; EURO, European regional office; EMRO, Eastern Mediterranean regional office; IP, internship programme; SEARO, South-east Asian regional office; WPRO, Western pacific regional office. * AMRO data was not included in the 2015 data set published by the WHO.

In April 2016, these were finally published:^7^ only 15% of interns at WHO headquarters were from developing countries; the two regional offices serving regions with the highest disease burdens—Africa and South-East Asia—offered in total <4% of all WHO internships (figure 1); and almost 60% of WHO Member States had no nationals participating in the entire programme throughout 2015 (table 1). These include large emerging economies such as Argentina and South Africa; as well as countries with beleaguered health systems recovering from recent or chronic health emergencies such as Liberia and Haiti. By the time of the 2016 WHA, WHO had provided Member States with a clear picture of the WHO internship programme's shortcomings. Accordingly, during the human resources Committee B discussion several Member States raised their concerns on the internship programme's findings—namely the under-representation of developing countries.^8^

**Table 1:** Table details countries with ‘0’ or less than 5 interns on WHO Internship Programme in 2015. Data compiled using WHO 2016 Human Resources Annual Report statistics and list of WHO Member States as of 2016.

*Countries not represented on WHO internship programme in 2015*		
Afghanistan	Bahrain	Cameroon
Albania	Barbados	Central African Republic
Algeria	Belize	Chad
Andorra	Bhutan	Comoros
Angola	Bolivia	Cook Islands
Antigua and Barbuda	Botswana	Costa Rica
Argentina	Brunei Darussalam	Croatia
Armenia	Burundi	Cuba
Azerbaijan	Cabo Verde	Cyprus
Bahamas	Cambodia	Czech Republic
Georgia	Israel	Libya
Grenada	Jamaica	Luxembourg
Guatemala	Kazakhstan	Madagascar
Guinea	Kiribati	Malawi
Guinea-Bissau	Kuwait	Maldives
Guyana	Kyrgyzstan	Malta
Haiti	Lao People's DR	Marshall Islands
Honduras	Latvia	Mauritania
Iceland	Lesotho	Mauritius
Iraq	Liberia	Micronesia (FS of)
Papua New Guinea	Seychelles	Tajikistan
Paraguay	Solomon Islands	TFYR of Macedonia
Peru	Somalia	Timor-Leste
Qatar	South Africa	Togo
Republic of Moldova	South Sudan	Tonga
Saint Kitts and Nevis	Sri Lanka	Trinidad and Tobago
Saint Lucia	St. Vincent	Tuvalu
Samoa	Suriname	United Arab Emirates
San Marino	Swaziland	Uzbekistan
Sao Tome and Principe	Syrian Arab Republic	Vanuatu
*Countries with <5 interns on WHO internship programme in 2015*		
Bangladesh	Cote d'Ivoire	Korea, Dem. People's Rep. Of
Belarus	Ecuador	Lebanon
Benin	Estonia	Malaysia
Bosnia and Herzegovina	Ghana	Mali
Bulgaria	Hungary	Moldova, Republic of
Burkina Faso	Indonesia	Mongolia
Chile	Iran	Morocco
Colombia	Ireland	Myanmar
Congo, DR	Jordan	Nepal
Congo, Republic of the	Kenya	New Zealand
Slovenia	Yemen	

## The implications

While the statistics concerning the internship programme are striking, the reasons for the inequitable representation of young professionals from developing countries are simple. Recruitment to the programme is ad hoc and not policy-driven; departments select candidates informally without reviewing all applications and therefore those with pre-existing connections are far more likely to be selected. Moreover, interns are not financially supported by WHO during their stay, even in expensive locations such as Geneva. This precludes candidates from Member States with significantly lower incomes participating in the programme. In so doing, the internship programme corrodes a vital relationship WHO seeks to cultivate with future health leaders in the developing world.

## Reform of the internship programme?

While staff appointments are subject to a formula that was last revised in 2003,^12^ the internship programme is subject to no such protocol. Regrettably, it is a lack of objective oversight that has let the programme's structural barriers go unaddressed. However, the attention and transparency now focused on the programme presents a unique opportunity for decision makers. In responding to the concerns of Member States at the 2016 WHA, both WHO's Head of Human Resources and the Assistant Director-General for General Management stated that with Member States’ support and funding, reform of the internship programme would be possible^8^—but what might this actually entail?

First, unguided, recruitment to the internship programme lacks transparency and favours candidates with pre-existing connections.^5^ While WHO reports that it is working to ‘promote internship opportunities’,^7^ the appointment of a designated staff officer with responsibility for programme administration will be necessary to ensure recruitment that is equitable. Second, internships are unpaid and there is no financial support offered to candidates from developing countries, precluding many from lower income states participating. WHO states that it is now referring prospective interns to ‘lists of scholarships’;^7^ however, these are notoriously difficult to locate, often limited in number and only available to nationals from certain countries. Provision of a nominal stipend to compensate interns—considered by Member States in January^13^ but absent from the WHO report in May^7^—would comprehensively eliminate this barrier at limited cost. The International Labour Organization has successfully implemented such a scheme. Finally, balanced distribution alone will not guarantee participants gain the experience and knowledge they need. Introduction of a semistructured curriculum would consolidate the programme's capacity building role and improve its credibility in the eyes of Member States and donors.

## Is it worth the effort?

In an increasingly complex and fragile global health environment, there are justifiably more immediate concerns for decision makers to prioritise. Yet, if global ambitions for building developing country capacity—as called for in the sustainable development goals—are to be credible, organisations such as WHO can ill afford to run a training programme that all but excludes young professionals from the developing world. With WHO's traditional leadership role under challenge, positive engagement with the next generation of public health professionals is a necessity.

By the time WHO reaches its centenary, many of today's young public health professionals *should* be at the centre of national and regional health security. Reform of the existing internship programme represents a time and resource-efficient way for WHO to use its own organisational resources and international reputation, to directly build equitable future global health capacity among junior public health professionals from the developing world. Recent progress suggests this argument is beginning to be heard; has the time come for WHO and its governing bodies to definitively act?
